# Efficacy and safety of *Guhong* injection for treating coronary microvascular disease: study protocol for a randomized controlled trial

**DOI:** 10.1186/s13063-019-3990-3

**Published:** 2020-01-13

**Authors:** Zhuang Jieqin, Liu Shuling, Cai Hairong, Dai Xingzhen, Chen Yanhong, Jin Zilin, Chen Bojun

**Affiliations:** 1 The Second Clinical Medical College of Guangzhou University of Traditional Chinese Medicine, Guangzhou, 510405 Guangdong Province China; 2 The Second Affiliated Hospital of Guangzhou University of Traditional Chinese Medicine, Guangzhou, 510006 Guangdong Province China

**Keywords:** *Guhong* injection, Coronary microvascular disease, Randomized controlled trial, Inflammatory response, Endothelial function

## Abstract

**Background:**

Coronary microvascular disease (CMVD) can be described as one of the cardiovascular diseases with normal coronary angiography but evidence of myocardial ischemia or microcirculatory lesions, often presenting as angina pectoris attacks. Coronary artery microtubular dysfunction is one of the pathogenic features of coronary heart disease, but its occurrence and development and the current CMVD-intervention therapy needs further research. Chinese traditional medicine (TCM) has advantages for the treatment of cardiovascular diseases. Hence, this article describes an ongoing randomized controlled clinical trial based on the theory of TCM for the purpose of evaluating the efficacy and safety of *Guhong* injection versus placebo in patients with CMVD.

**Methods/design:**

This is a multicenter, randomized, parallel-arm, open-label, double-blind, placebo-controlled clinical trial. A total of 260 eligible patients will be allocated and randomly assigned, in a ratio of 1:1, to either the experimental group or the control group. The treatment course is 10 consecutive days, and with an 8-week follow-up. The primary outcome is therapeutic efficacy. Secondary outcomes include the quantitative score of TCM syndromes (a series of TCM symptoms and signs of coronary heart disease), the average frequency of anginal attacks, electrocardiogram (ECG) changes, inflammatory response, endothelial function indicators and myocardial metabolites.

**Discussion:**

This trial is strictly designed in accordance with principles and regulations issued by the China Food and Drug Administration (CFDA). The results should provide high-quality evidence on the efficacy and safety of *Guhong* injection in the treatment of CMVD.

**Trial registration:**

Chinese Clinical Trials Registry, ID: ChiCTR1900022902. Registered on 27 April 2019.

## Background

Coronary microvascular disease (CMVD) is a clinical syndrome with objective evidence of exertional angina or myocardial ischemia caused by structural or functional abnormalities of the precoronary arteries and arterioles under the influence of multiple pathogenic factors. In the past 20 years, a large number of studies have shown that coronary microcirculatory structural and functional abnormalities can be found in non-occlusive coronary artery diseases, but also in people with high risk factors for coronary heart disease, as well as in cardiomyopathic and occlusive coronary artery diseases, which have important predictive and therapeutic target values [1, 2]. Currently, there are still no large-sample epidemiological data on CMVD, but some clinical studies with small samples have shown that the incidence of CMVD is up to 45–60% in patients with myocardial ischemic symptoms but coronary angiography showing non-obstructive lesions, and the major cardiovascular events and all-cause mortality in these patients are significantly higher than in the control group [3]. It is currently generally believed that the pathogenesis of CMVD is complex. Endothelial injury, inflammatory factors, various vascular substances and microembolism may all lead to changes in microvascular structure and function, and the pathophysiological mechanism of different types of CMVD varies in clinical practice [4–6]. At present, treatment regimens using western medicine mainly include anti-thrombotic agents, lipid regulation, plaque stabilization and vascular dilation, etc., but these methods are not always successful and improved treatment remains an urgent problem to be addressed in clinical practice [7], Many patients still have repeated anginal attacks under the current drug treatment regimen, causing psychological anxiety and restlessness, which has a great impact on life and work.

TCM has the advantage of its multi-target and multi-link therapeutic effects and less adverse reactions than western therapies, especially for the treatment of cardiovascular and cerebrovascular diseases, and also has a long history and has been widely studied and its benefits confirmed. TCM treatment depends on different pathogenic mechanisms of disease but blood stasis is generally considered as one of the most important for coronary heart disease, so promoting blood circulation and removing blood stasis is an extremely important treatment goal [8]. In recent years, TCM research in the prevention and treatment of CMVD has shown good application prospects, but this is still not universally agreed treatment.

Redflower is one of the most famous TCM and is considered to be essential for promoting blood circulation and removing blood stasis [9]. *Guhong* injection is made with redflower extracts and acetylglutamide; of them, redflower extracts contain active ingredients such as redflower glycosides and redflower yellow pigment (Table 1). Clinical pharmacology research results show that *Guhong* injection can inhibit platelet aggregation, prevent thrombosis, cause expansion of the coronary arteries, reduce myocardial oxygen consumption, improve myocardial microcirculation, alleviate ischemia-reperfusion injury, and reduce anti-oxygen free radicals [10]. Accordingly, this study intends to use *Guhong* injection by peripheral intravenous drip as the treatment of CMVD to demonstrate its efficacy and safety, and to explore its mechanism of action from the perspective of the inflammatory response and endothelial function, so as to provide a basis for the treatment of coronary artery microvascular dysfunction using integrated TCM and western medicine.

**Table 1:** Pharmacological effects of each ingredient in *Guhong* injection

Ingredient	Pharmacological effects
Redflower glycosides	Resist myocardial ischemia, resist platelet aggregation
Redflower yellow pigment	Protect heart muscle, resist oxidation, resist inflammation
Acetylglutamide	Activate nerve cell

## Methods/design

### Study design

**Fig. 1:**
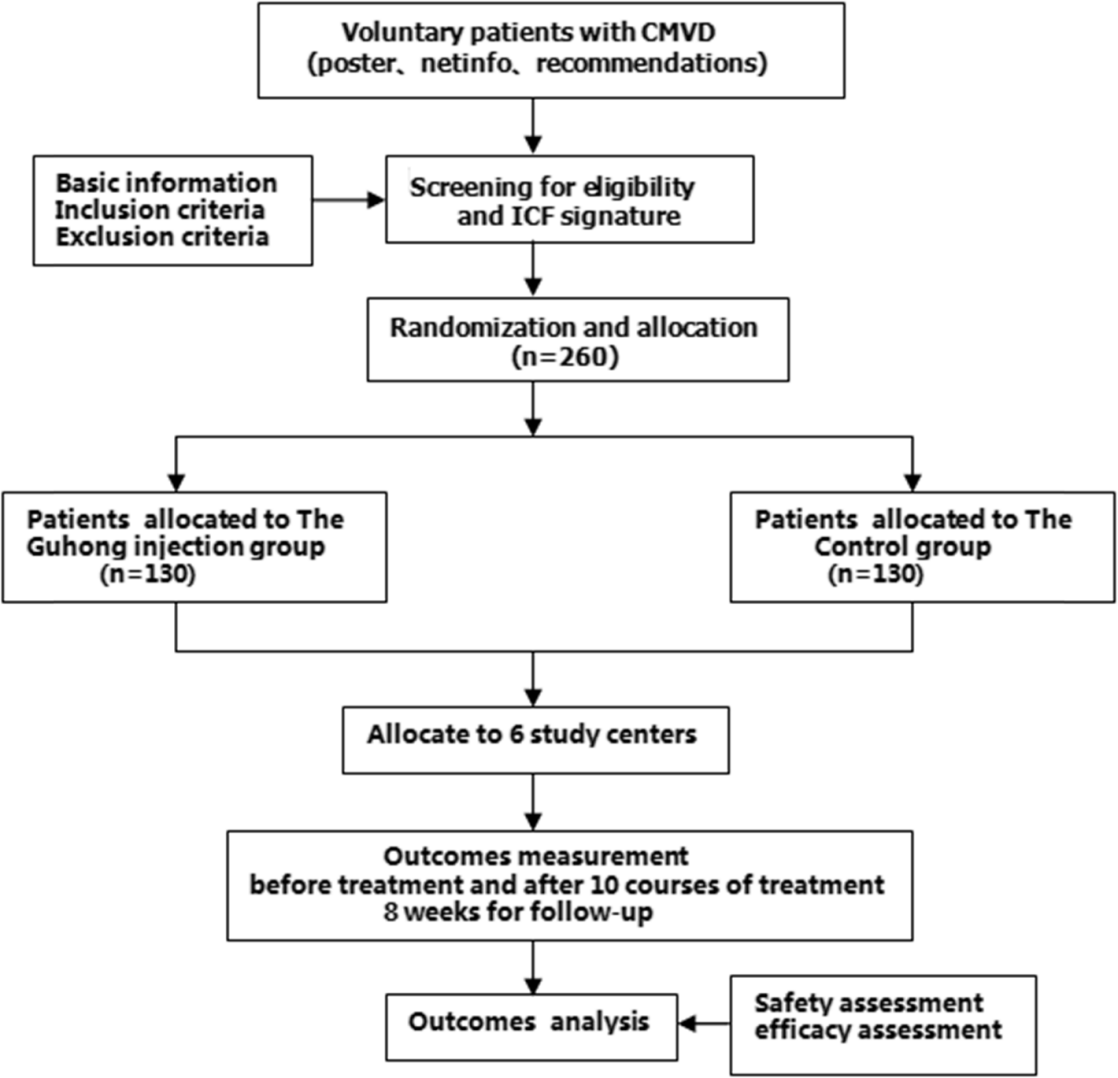
Study flow chart. *ICF* informed consent form

This study is a multicenter, parallel, prospective, double-blind, randomized, placebo-controlled clinical trial. A total of 260 patients will be enrolled and randomly divided into the treatment and placebo groups. All patients will receive 10 consecutive days of treatment and follow-up for 8 weeks. Efficacy and safety data will be collected throughout the study. The study flow chart is shown in Fig. 1.

### Ethics

This trial has been successfully registered in the China Clinical Trial Registry (ID: ChiCTR1900022902). Additionally, the study will be strictly conducted in accordance with the Helsinki Declaration, Good Clinical Practice (GCP) guidelines and national laws. The research protocol, informed consent, and recruitment posters were reviewed and approved by the Ethics Committee of the Guangdong Provincial Hospital of Traditional Chinese Medicine (No. BF2018–180-01). The researchers in all six trial centers are well-trained and qualified medical staff. All eligible patients will be fully informed and understand about this protocol and will sign an informed consent form (ICF) prior to participation. All participants can receive all examinations and treatments for free, and their privacy, data and security will be securely protected.

### Participants and recruitment

We will recruit patients who meet the criteria for inclusion by advertising on hospital notice boards, posting recruitment information in network communication groups or by being recommended by outpatient and inpatient physicians. A total of 260 eligible participants will be recruited in the following six hospitals: (1) Guangdong Provincial Hospital of Traditional Chinese Medicine, (2) Guizhou Provincial Hospital of Traditional Chinese Medicine, (3) Second Affiliated Hospital of Guizhou University of Traditional Chinese Medicine, (4) Foshan Traditional Chinese Medicine Hospital, (5) Shenzhen Longgang District Hospital of Traditional Chinese Medicine and (6) Yangjiang People’s Hospital. These six trial centers are all first-class hospitals in China, with advanced cardiovascular interventional departments, excellent medical teams and many inpatient beds. Especially the Guangdong Provincial Hospital of Traditional Chinese Medicine, which has the largest number of outpatients among the TCM hospitals in China and rich experience and achievements in the research of integrated Chinese and western medicine. So, the first center will recruit 80 patients, with 36 for each of the remaining centers.

### Inclusion criteria

For inclusion, participants should fulfill all the following criteria:

1. Coronary angiography or coronary computed tomography (CT) indicating the absence of unstable plaques, or coronary stenosis < 50% and the presence of clinical symptoms of stable angina pectoris [11].
2. Aged between 35 and 75 years, regardless of gender
3. Willingness to participate in the trial and to sign the ICF, with high degree of compliance and a cooperative attitude

### Exclusion criteria

Participants with any of the following conditions will be excluded:

1. Significant psychopathology, pregnant or lactating women
2. Patients with a history of myocardial infarction, heart failure, stroke, arterial dissection, arterial embolization, tumors, severe hematologic diseases, endocrine diseases or pneumonia and other infections
3. Patients with liver and kidney dysfunction (expression of aminotransferase (ALT) and aspartate aminotransferase (AST) of 1.5 times higher than the normal upper limit, abnormal serum creatinine, positive urinary protein qualitative test)
4. Patients who are allergic to *Guhong* injection
5. Participation in other clinical trials and using the actual experimental drugs within the preceding 3 months
6. Patients who are not considered by the researchers to meet the inclusion criteria

### Withdrawal criteria

The withdrawal criteria include the following:

1. Experiencing serious complications or rapid deterioration of the condition during the trial, including anginal attack frequency increasing significantly, myocardial ischemia continuing to worsen, myocardial infarction, arrhythmia and other changes
2. Serious adverse events occurring, which should lead to treatment being stopped according to the physician’s judgment
3. Participants with important deviations in the implementation of the program, such as poor compliance and difficulty in evaluating drug effects
4. Quitting the clinical trial voluntarily

### Sample size

A previous study for *Guhong* injection in the treatment of angina pectoris [12] showed that the effective rate of the conventional treatment group (33 cases) was 81.82%, while that of the *Guhong* injection plus conventional treatment group (33 cases) was 93.94%. According to the literature, it was assumed that the efficiency rate of the *Guhong* injection group was from 81.82–93.94%, and the sample size was calculated based on the parameter *α* = 0.05 (bilateral test) and *β* = 0.2. By comparing the effectiveness rate of the two groups according to the following sample-size estimation formula:

$$ \mathrm{N}=\left[{\uppi}_{\mathrm{t}}\ \left(1-{\uppi}_{\mathrm{t}}\right)/\mathrm{k}+{\uppi}_{\mathrm{c}}\ \left(1-{\uppi}_{\mathrm{c}}\right)\right]\left(\ {\upmu}_{1-\upalpha /2}+{\upmu}_{1-\upbeta}\right)2/\left({\uppi}_{\mathrm{t}}-{\uppi}_{\mathrm{c}}\right), $$

we calculated that 111 patients should be recruited for each group. Taking into consideration an attrition rate of < 15%, the eligible participants in each group should be > 130. Ultimately, we determined the final sample size to be 130 (*N* = 260 in total).

### Randomization and blinding

A total of 260 participants will be randomly assigned to the *Guhong* group and the control group in a ratio of 1:1 by the method of using 260 opaque envelopes. Each envelope will be stuffed with a piece of paper. There are 260 pieces of papers in total, half of which have the words: “20 ml *Guhong* injection plus 250 ml 0.9% sodium chloride, ivd, qd” written on them and the other half have the words “270 ml 0.9% sodium chloride, ivd, qd” written on them. After all the opaque envelopes have been stuffed and sealed, they will be mixed evenly and distributed to each trial center.

When a sub-center accepts an eligible participant, the investigators will write the baseline information (including the subject’s hospital ID number, name, age and gender) on the cover of the envelope. Both investigators and participants will be blinded. Only drug administrators and dispensing nurses are responsible for opening the envelope to know the group allocation and perform the interventions according to the written instructions on the paper, but they won’t be involved in recruitment, recommendations, data collection and data analysis. Both the *Guhong* injection and placebo are administered using a photophobic infusion set to avoid the subjects being aware of the group information. All investigators, outcome assessors and data analysts will be blinded to collect and summarize the data which is only based on a subject’s baseline information until the completion of the visit and analysis.

### Emergency unblinding

If an emergency occurs, the patient group assignment should be unblinded and known to the drug administrators or dispensing nurses, and corresponding emergency measures will be taken. Researchers will report this special situation to the center’s principal within 24 h. At the same time, the cause of unblinding, the time of the emergency, the solution and the result must be filled in the case report form (CRF). Once the participant ‘s allocated intervention during the trials has been revealed, the case will be withdrawn and the data will be recorded on day 10 of the trial evaluation.

### Unblinding after the study

When all trials are completed and all data have been locked, the unblinding process will be conducted in the presence of the researchers.

### Interventions

On the basis of conventional western medicine treatment, the *Guhong* group will be given a 20-ml intravenous infusion of *Guhong* injection that was diluted with 250 ml 0.9% sodium chloride injection once a day, while the control group will be given 270 ml 0.9% sodium chloride injection as placebo treatment once a day. (*Guhong* injection is provided by China tonghua guhong pharmaceutical co. Ltd. The production batch number is 20190306 and each bottle has a capacity of 5 ml. The drug distributed to each center will be labeled with the statement “for trial only” and the information of the name, dosage, dosing plan, indications, storage conditions, expiratory date, manufacturer and so on. Each center has an independent manager responsible for receiving, handling, storing and distributing the medications.) The course of treatment is 10 days. On the day of enrollment and at the end of treatment, the clinical symptoms and signs of angina pectoris will be recorded, the quantitative table of TCM syndrome will be scored, the electrocardiogram (ECG) will be examined, and the indicators of inflammation, endothelial function, myocardial metabolites and related biochemical tests will be conducted. Moreover, patients will be followed up for 8 weeks after the end of treatment, including any improvement in angina pectoris and the TCM syndrome quantitative score. During the entire study period, participants will be visited for a total of four times by the investigators. Specific research process details are provided in Table 2.

**Table 2:**
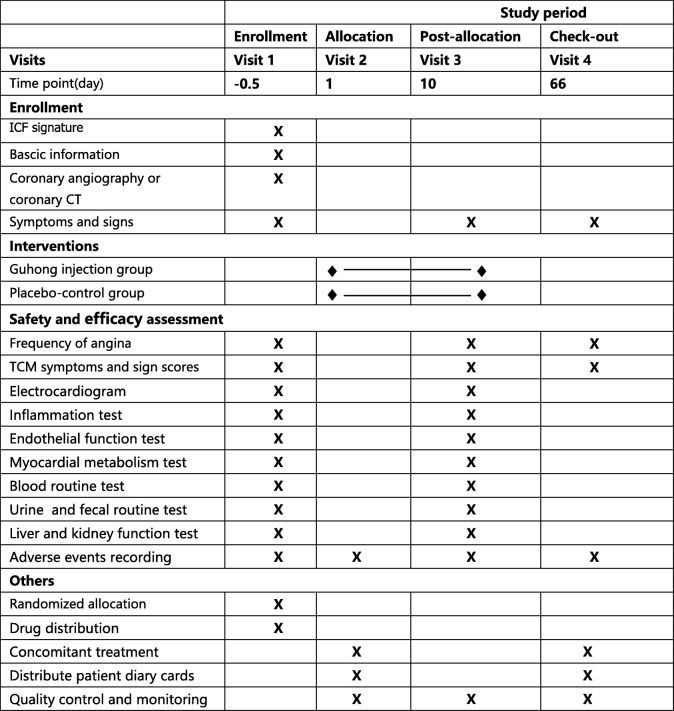
Study schedule for patients

### Concomitant treatments and forbidden drugs

During the study period, participants with other diseases, such as hypertension, diabetes, hyperlipidemia, etc., are allowed to continue any corresponding treatment using western medicine. Also the name, dosage and duration of any concomitant medication must be carefully recorded in the CRF, and any other western medicine or Chinese medicine that may affect the research results will be prohibited. Once banned drugs are used, patients will be removed from the study and their data will not be used, in accordance with the signed ICF.

### Efficacy assessment

#### Primary outcome

The criteria for therapeutic efficiency evaluation is based on the principles for clinical research reports on cardiovascular drugs formulated by the National Health and Pharmaceutical Bureau of China [13] by comparing the anginal attack frequency and ECG changes before and after treatment in the two groups of participants. Specific evaluation criteria are shown in Table 3.

**Table 3:** Evaluation criteria on the therapeutic efficiency

Three-graded criteria	Detailed description	Classification
Significant improvement	Anginal attacks disappeared or significantly improved, decreased by 80%; electrocardiogram (ECG) returned to normal or substantially normal	Significantly effective
Improvement	Anginal attacks improved or the frequency of anginal attacks decreased by 50–80%; the descending ST segment of the ECG rose ≥ 0.05 mV, and the ascending ST segment fell ≥ 0.05 mV, but still did not return to normal; or the T wave of the main ECG lead changed from flat to upright; or the T wave changed from inverted to shallow by more than 50%	Effective
No improvement	No change or even aggravation of anginal attacks, no decrease or even increase of the frequency of angina pectoris attacks, no change or even aggravation of ECG ST-T segment	No effect

#### Secondary outcomes

Secondary outcomes include changes in TCM symptoms and signs scores (Table 4), the average frequency of angina pectoris, the ECG, the indicators of inflammation, endothelial function and myocardial metabolites.

**Table 4:**
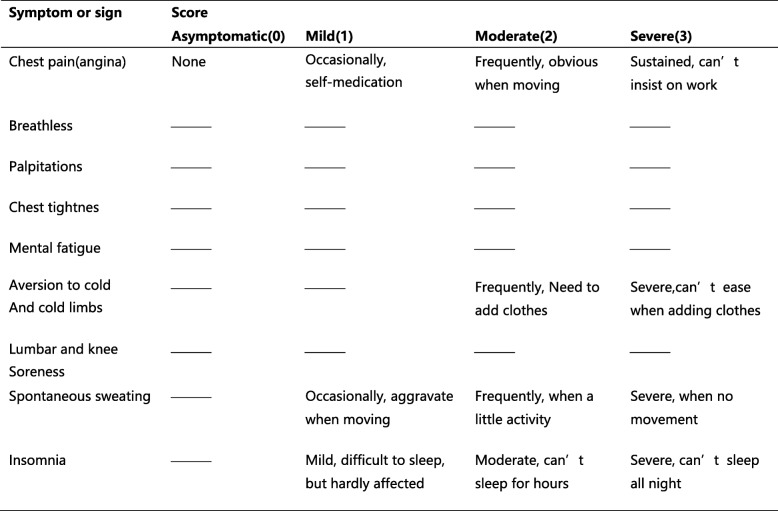
Symptom and sign scoring system

#### Change in scores of TCM symptoms and signs

The TCM syndromes scoring system used in the study follows the guidelines of clinical research on the treatment of coronary heart disease (chest pain) with new Chinese medicine [14], in which all symptom and sign scores are graded (Table 4). A total of nine kinds of symptoms include: chest pain, chest tightness, breathlessness, palpitations, mental fatigue, aversion to cold, and cold limbs, lumbar and knee soreness, spontaneous sweating, and insomnia. The score is 0 points for asymptomatic, 1 point for mild, 2 points for moderate and 3 points for severe. The total score ranges from 0 to 27. The lower the score, the milder the symptoms.

#### The average frequency of angina pectoris

The average frequency of angina pectoris from the first day of enrollment to the end of treatment and at 8 weeks of follow-up will be recorded in the CRF. Each participant will be instructed to record any change in their symptoms of angina pectoris in their patient diary daily.

#### Indicators of inflammation, endothelial function and myocardial metabolism

Indicators of inflammation, endothelial function and myocardial metabolism of participants before and after treatment will be tested in this study. Inflammatory indicators include hypersensitive c-reactive protein (hs-CRP), interleukin-6 (IL-6)、tumor necrosis factor-alpha (TNF-α). Endothelial function indices include nitric oxide (NO), endothelin-1 (ET-1)、and thromboxane A2 (TXA2). Myocardial metabolism indicators include cardiac troponin I (cTnI) and creatine kinase isoenzyme (CK-MB). Inflammatory reaction plays a significant role in the occurrence and development of the coronary heart disease symptom angina pectoris. TNF-α is the initiation factor of the inflammatory response, which stimulates the synthesis and release of pro-inflammatory response factors (IL-6 and CRP), leading to the aggravation of the inflammatory response and the formation of thrombus [15, 16]. NO and ET-1 are the two most important vasoactive factors and the sensitive indicator of endothelial dysfunction in the human body [17], NO can relax blood vessels [18], while ET-1 can promote vasoconstriction [19]. After vascular endothelial injury, TXA2 will promote platelet aggregation and vasoconstriction [20]. As for cTnI and CK-MB, they are ideal metabolites for early myocardial injury [21].

#### Safety evaluation

Liver and kidney function, routine blood tests, routine urine tests, routine fecal tests and an ECG before and after treatment will be used to assess for drug safety and adverse reactions.

#### Data management and statistical analysis

The Drug Clinical Research Center of Guangzhou University of TCM will be responsible for the data statistical analysis. Two independent data administrators are in charge of reading the CRFs and recording data on the EpiData 3.1 software. Clinical research assistants (CRA) and investigators will re-check and review the accuracy and consistency of the data. After entering and reviewing, the database will be locked and the statistical analysis will be performed only with the permission of the sponsor, the principal investigator and the investigators.

The full analysis set (FAS) is the primary analysis set, in which patients should be dosed with *Guhong* injection or placebo for 10 days with clinical observations recorded in the study. All subjects without any major protocol deviations will be included in the per-protocol set (PPS). Efficacy assessment will be performed through FAS and PPS. Safety evaluation will be conducted for those who have been treated at least once, which is defined as the safety set (SS).

All continuous data and normal distribution data are represented by mean ± standard deviation, with median and range for non-normal data. Classified data are expressed in frequency counts and percentages. Baseline balance between groups will be determined by a chi-square test or analysis of covariance (ANOVA). All the indicators of anginal attack frequency, inflammation, endothelial function and myocardial metabolites will be estimated and compared using the log-rank test. A Kaplan-Meier survival curve will be constructed, and the median time will be provided separately for each group with a two-sided 95% confidence interval. ANOVA and Bonferroni methods will be used to compare the TCM symptom and sign scores of the two groups. All collected data will be processed by professional statisticians using SAS 9.2 software; a two-sided *P* value of < 0.05 is considered statistically significant.

#### Quality control and monitoring

All CRFs are designed in strict accordance with relevant requirements of GCP, which will be filled out by trained researchers to ensure consistency and reduce bias. If there is any ambiguity in the completion process, the results will be determined by the team members of the whole center after discussion. Each trial center has a project leader to control the quality, and all survey researchers are trained to be qualified and familiar with the process. In the process of clinical research, measures should be taken to ensure patient compliance according to the possible causes of loss to follow-up, and designated, special cardiologists will visit each center regularly to check the original documents and CRFs, and supervise the study to ensure that it complies with the protocol. Any shortcomings or problems found by the inspectors should be improved.

#### Interim analyses and stopping guidelines

When the trial is nearly halfway through, an interim analysis of the collected data will be performed. Only the sponsor and the principal investigator will have access to these interim results and decide whether or not to make the final decision to terminate the trial. The guidelines for stopping the trial are listed as follows: (1) The efficacy and safety of the studied drug have been confirmed; (2) The expected inter-group effect difference cannot be achieved and (3) There was an intolerable adverse drug reaction.

## Discussion

Many studies have confirmed that the coronary microcirculation plays an important role in regulating coronary blood flow and myocardial perfusion [22]. However, it cannot be easily examined in coronary angiography, so it is easy to neglected it in clinical work. Therefore, while we focus on the prevention and treatment of coronary artery disease, we should pay enough attention to CMVD because it affects a major section of the coronary heart disease population. To date, there are no drugs or methods on the treatment of CMVD that have been proved to be effective in extensive clinical studies [23]. However, TCM has been used for cardiovascular and cerebrovascular diseases for thousands of years in China. Moreover, many Chinese medicines have been developed by combining the historical therapeutic experience of TCM with modern pharmacological research, and their efficacy and safety have been repeatedly confirmed by studies. However, it is a pity that many of these studies have limitations in their general quality and are of low quantity [24], so it is hard to popularize TCM and apply it more globally.

As a Chinese medicine developed by many well-known domestic pharmaceutical groups, *Guhong* injection has been proved to be effective and has been used for the treatment of cardiovascular and cerebrovascular diseases in clinical practice for many years. In order to demonstrate the efficacy and safety of *Guhong* injection for CMVD, and to investigate its mechanism of action from the perspective of inflammation and endothelial function, we applied for this multicenter, double-blind, placebo-controlled randomized clinical trial in accordance with the Consolidated Standards of Reporting Trials (CONSORT) guidelines and the “One study, one primary outcome” clinical trial methodology published by the CFDA [25] for the purpose of obtaining high-quality evidence for clinical extension. Moreover, the protocol presents a detailed and practical methodology for future clinical trials of developing TCM (Additional file 1).

## Trial status

The study is currently in the process of continuing to enroll participants in six trial centers. The protocol version number is 1.0, dated 1 November 2018. Our recruitment period will be from 19 December 2018 to 19 December 2020.

## Supplementary information

**Additional file 1:** Standard Protocol Items: Recommendations for Interventional Trials (SPIRIT) 2013 Checklist: recommended items to address in a clinical trial protocol and related documents.

## Data Availability

All data will be made available

## Abbreviations

ALT: Alanine aminotransferase
AST: Aspartate aminotransferase
CFDA: China Food and Drug Administration
ChiCTR-IPR: China Clinical Trial Registry
CK-MB: Creatine kinase isoenzyme
CMVD: Coronary microvascular disease
CONSORT: Consolidated Standards of Reporting Trials
CRA: Clinical research assistants
CRF: Case report form
CRO: Contract Research Organization
cTnI: Troponin I
ET-1: Endothelin-1
FAS: Full analysis set
GCP: Good Clinical Practice
hs-CRP: Hypersensitive c-reactive protein
ICF: Informed consent form
IL-6: Interleukin-6
PPS: Per-protocol set
SS: Safety set
TCM: Chinese traditional medicine
TNF-α: Tumor necrosis factor-alpha
TXA2: Thromboxane A2
